# Hospital readmissions for COPD: a retrospective longitudinal study

**DOI:** 10.1038/s41533-017-0028-8

**Published:** 2017-04-27

**Authors:** Timothy H. Harries, Hannah Thornton, Siobhan Crichton, Peter Schofield, Alexander Gilkes, Patrick T. White

**Affiliations:** 1 King’s College London, King’s Health Partners, Division of Health and Social Care Research, London, SE1 3QD UK; 2 Centre for Academic Primary Care, NIHR School for Primary Care Research, School of Social and Community Medicine, University of Bristol, Canynge Hall, 39 Whatley Road, Bristol, BS8 2PS UK

## Abstract

Prevention of chronic obstructive pulmonary disease hospital readmissions is an international priority aimed to slow disease progression and limit costs. Evidence of the risk of readmission and of interventions that might prevent it is lacking. We aimed to determine readmission risk for chronic obstructive pulmonary disease, factors influencing that risk, and variation in readmission risk between hospitals across 7.5 million people in London. This retrospective longitudinal observational study included all chronic obstructive pulmonary disease admissions to any hospital in the United Kingdom among patients registered at London general practices who had emergency National Health Service chronic obstructive pulmonary disease hospital admissions between April 2006 and March 2010. Influence of patient characteristics, geographical deprivation score, length of stay, day of week of admission or of discharge, and admitting hospital, were assessed using multiple logistic regression. 38,894 chronic obstructive pulmonary disease admissions of 20,932 patients aged ≥ 45 years registered with London general practices were recorded. 6295 patients (32.2%) had at least one chronic obstructive pulmonary disease readmission within 1 year. 1993 patients (10.2%) were readmitted within 30 days and 3471 patients (17.8%) were readmitted within 90 days. Age and patient geographical deprivation score were very weak predictors of readmission. Rates of chronic obstructive pulmonary disease readmissions within 30 days and within 90 days did not vary among the majority of hospitals. The finding of lower chronic obstructive pulmonary disease readmission rates than was previously estimated and the limited variation in these rates between hospitals suggests that the opportunity to reduce chronic obstructive pulmonary disease readmission risk is small.

## Chronic lung disease: Lower risk of readmission for London-based patients

A managed reduction of hospital readmissions for London-based chronic lung disease patients may not be needed. Preventing hospital readmissions for patients with chronic obstructive pulmonary disease (COPD) is a key priority to improve patient care and limit costs. However, few data are available to determine and ultimately reduce the risk of readmission. Timothy Harries at King’s College, London, and co-workers conducted a longitudinal study incorporating all COPD admissions into UK hospitals for 20,932 patients registered at London general practitioners between 2006 and 2010. They found that 32% of patients were readmitted within a year, 17.8% within 90 days and 10% within 30 days. Neither age nor geographical deprivation were useful predictors of readmission. These represent lower than estimated levels of readmission, suggesting there may be fewer opportunities to reduce the risk of readmission further.

## Introduction

Chronic obstructive pulmonary disease (COPD) is a leading cause of emergency hospital medical admissions and readmissions worldwide.^1, 2^ Preventing COPD readmissions following an exacerbation has been identified as an international priority to limit the physical deterioration of patients and to contain costs.^3, 4^ In an effort to curb hospital readmissions health service payments to United Kingdom (UK) National Health Service (NHS) hospitals for patients readmitted within 30 days of discharge have been restricted since 2011 under the Payment by Results scheme.^5, 6^ This is especially the case for those readmissions judged to be avoidable. In COPD the rate of readmissions within 30 days has been used as a marker of quality of care within the NHS.^7^ COPD is an ambulatory care sensitive condition (ACSC) for which it is considered that hospital admission may be avoided by effective interventions in primary or preventative care.^8^ Reductions of up to 18% in emergency admissions for ACSCs have been estimated to be achievable.^9^ ACSCs were included as an outcome indicator upon which NHS hospital reimbursement was based as recently as 2014–2015.^10^ The reliability of this measure is doubtful as the true proportion of avoidable readmissions is not known.^11^ Evidence is lacking on the effectiveness of case-based interventions in reducing admission risk.^12^ Smoking is a known risk factor for the development of COPD and is a predictor of COPD exacerbations.^13, 14^

The rate of emergency admissions within the NHS in England between 2000 and 2012 increased by 27%, coinciding with approximately a 24% decrease in general and acute hospital bed numbers over the same period.^15^ The all-cause 30-day readmission rate within the NHS in England between 2004 and 2010 was 7.0% with a gradual increase of 0.01% per month.^16^ A meta-analysis of international studies identified that fewer than one in four 30-day readmissions was likely to have been preventable.^17^

Patient level factors have been identified as determinants of COPD readmission risk. These include a history of previous hospital admission, poor performance status of the patient, COPD disease severity, number of comorbidities, lower reported health status and quality of life score and dyspnoea on admission.^18–21^ The influence of service level predictors on the risk of COPD hospital admission and readmission is unclear. Within the NHS the rate of COPD admissions remained stable between 2001 and 2010,^22^ and variation in primary care services for COPD has not been shown to be associated with risk of COPD admission.^23^ A series of influential audits of COPD hospital care by the UK Royal College of Physicians (RCP) found that hospital resource and organisational factors were not associated with the rate of COPD readmissions and did not account for variation in COPD readmission risk between hospitals.^24^ No association was found between type of admitting physician, hospital admissions policy, size of hospital respiratory unit, use of an early discharge scheme, length of hospital stay or presence of pneumonia and risk of COPD readmission.^25–27^

COPD readmission rates vary between countries. Within the UK the RCP audits recorded 90-day COPD readmission rates of 31–35%.^19, 24, 28, 29^ In other parts of the world 30-day readmission rate is the more commonly used outcome measure. 30-day COPD readmission rates within the US have been reported to be as low as 5.6% and as high as 20%.^30–32^ Observational data from New Zealand found a 30-day COPD readmission rate of 6.7%.^33^

The extent to which COPD readmissions can be prevented is unknown. In treatment naïve patients there is good evidence for the effectiveness of medications in reducing exacerbation and admission risk.^34^ In the UK, the adoption of active prescribing of medications for COPD has been so great that there is now widespread over-prescribing of high dose inhaled corticosteroids in combination with long-acting inhaled beta-agonists.^35^ Although studies of the effect of post-discharge care schemes suggest that COPD readmission risk may be modifiable,^34, 36, 37^ these are tempered by contradictory findings from other initiatives that have been unsuccessful in reducing COPD readmissions.^38, 39^ Despite efforts to reduce the number of admissions, total hospitalisations and emergency department visits for COPD have not decreased over the past decade.^40^ Evidence of variation in readmission risk between hospitals may provide an opportunity for hospital managers and those commissioning services to improve the quality of care of COPD patients in hospitals with higher readmission risk.

This study aimed to describe the readmission risk of COPD hospital admission of all patients registered at all London general practices between 2006 and 2010. It sought to determine predictors of COPD readmission and the variation in risk of COPD readmission between different hospitals.

## Results

COPD admissions (38,894) from 20,932 patients aged ≥ 45 years registered with general practices in London were recorded between 1st April 2006 and 31st March 2010. These 20,932 patients each had an index COPD admission between 1st April 2006 and 31st March 2009.

Among the index COPD admissions 15,907 (76%) occurred on a weekday and 5025 (24%) at the weekend. 18,449 (88.1%) discharges occurred on a weekday and 2483 (11.9%) at the weekend. 2257 (10.8%) patients were admitted for <1 day. Index admissions had a mean (SD) length of stay (LOS) of 7.8 (11.3) days. Median (IQR) LOS was 5 (2–9) days. 1381 (6.6%) patients died during the index admission. 19,551 patients were at risk of readmission in the subsequent year. The outcome of admission was not available for 2% of patients.

Patients who were readmitted with COPD within 1 year (6295) were slightly more deprived and a slightly higher proportion of them were male than those patients who were not readmitted with COPD within 1 year (13,256) (Table 1).

**Table 1:** Comparison between patients who were readmitted with COPD within 1 year (6295) of the index COPD admission and those patients who were not readmitted within 1 year (13,256), 2006–2010

Patient characteristic		Mean (SD)	Difference between means (±95% CI)
Patient age	Readmitted	72.5 (10.3)	0.2 (−0.17 to 0.48)
(years)	Not readmitted	72.3 (11.1)	
Deprivation	Readmitted	29.2 (13.1)	1.2 (0.89 to 1.68)
score (IMD)	Not readmitted	28.0 (13.3)	
Males (%)	Readmitted	53.6	
	Not readmitted	51.5	

*CI* confidence interval, *IMD* index of multiple deprivation, *SD* standard deviation, *df* degrees of freedom

Difference between proportion of males (%): *χ* ^*2*^(1) = 8.15, *p* = 0.004

### Risk of COPD readmission

6295 patients (32.2%) had at least one COPD readmission within 1 year. In their first COPD readmission 164 patients (0.8%) were readmitted on the same day, 1993 patients (10.2%) were readmitted within 30 days, 3471 patients (17.8%) were readmitted within 90 days and 4698 patients (24.0%) were readmitted within 182 days.

### Patient factors associated with COPD readmission

Very weak correlations were found between patient age and risk of COPD readmission within 30 days (*r* = 0.034, *p* < 0.0001) and between patient age and risk of COPD readmission within 90 days (*r* = 0.032, *p* < 0.0001). Very weak correlations were found between patient Index of Multiple Deprivation (IMD) score and risk of COPD readmission within 30 days (*r* = 0.017, *p* = 0.015) and between patient IMD score and risk of COPD readmission within 90 days (*r* = 0.028, *p* < 0.0001).

### Influence of LOS on COPD readmission risk

Patients whose index COPD admission had a LOS of 3–5 days had a lower risk of COPD readmission within 30 days compared to patients whose index COPD admission lasted for 2 days or less (Table 2). Patients whose index COPD admission had a LOS of greater than 9 days had a greater risk of COPD readmission within 90 days compared to those patients whose index COPD admission lasted for 2 days or less.

**Table 2:** Comparison of the risk (odds ratio ± 95% CI) of COPD readmission within 30 days and 90 days, dependent on the LOS of the index COPD admission. Multiple logistic regression adjusted for patient sex, age, and deprivation score

	Readmission rate within 30 days	Readmission rate within 90 days
LOS** ≤ **2 days (Reference)	10.4%	16.6%
LOS 3–5 days	9.4% OR: 0.87 (0.77–0.99)	17.6% OR: 1.06 (0.96–1.17)
LOS 6–9 days	9.9% OR: 0.93 (0.82–1.07)	18.1% OR: 1.09 (0.98–1.22)
LOS > 9 days	11.1% OR: 1.03 (0.91–1.17)	19.3% OR: 1.17 (1.05–1.30)

### Influence of day of the week of admission and discharge on COPD readmission risk

Risk of COPD readmission within 30 days and within 90 days was unaffected either by the day of the week on which the index COPD admission had occurred or by the day of the week on which the patient was discharged from their index COPD admission.

### Analysis of COPD readmission rate by hospital

**Fig. 1:**
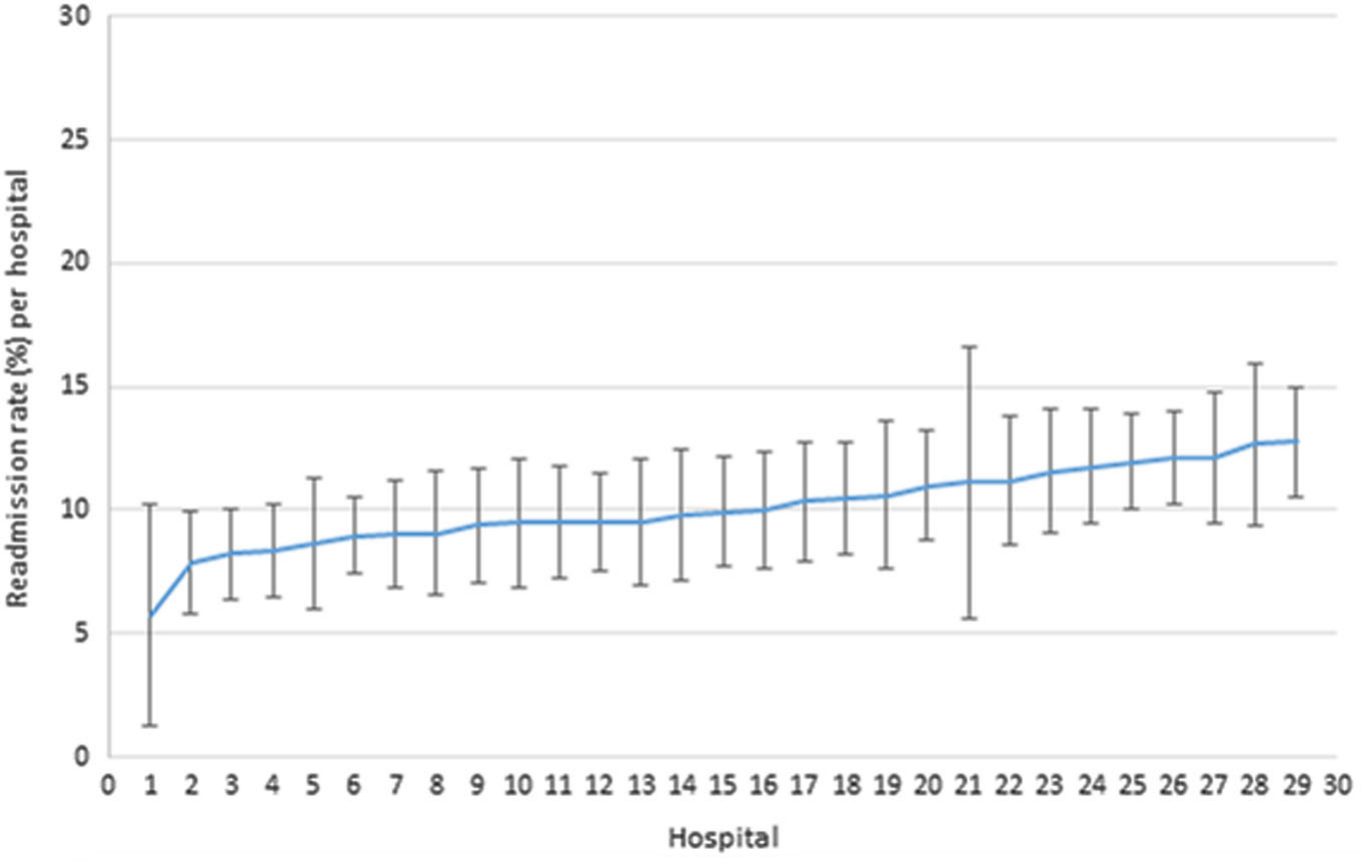
COPD readmission rates to hospitals within 30 days. Readmission rates (±95% CI) within 30 days of all COPD patients admitted (18,848) to included English hospitals between 2006 and 2010, shown in ascending order and adjusted for clustering, age and sex of patients

**Fig. 2:**
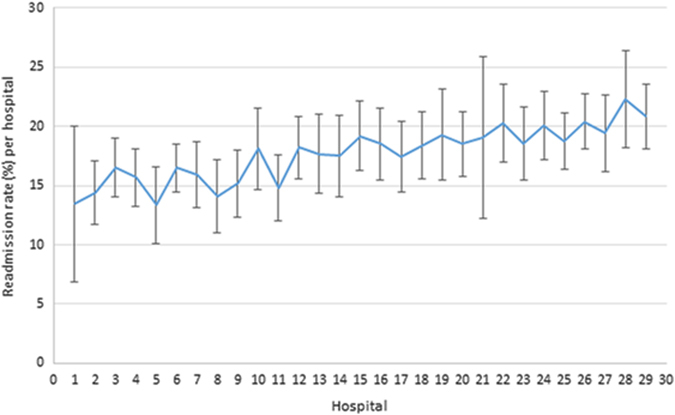
COPD readmission rates to hospitals within 90 days. Readmission rates (±95% CI) within 90 days of all COPD patients admitted (18,848) to included English hospitals between 2006 and 2010, and adjusted for clustering, age and sex of patients

The mean COPD readmission rate within 30 days to any hospital, among those hospitals which had received index COPD admissions, varied between 5.8% (95% CI: 1.3 to 10.2) and 12.7% (95% CI: 10.5 to 15.0) after adjustment for patient age, sex and the effect of clustering of patients within hospitals (Fig. 1). The mean COPD readmission rate within 90 days to any hospital, among those hospitals which had received index COPD admissions, varied between 13.3% (95% CI: 10.1 to 16.6) and 22.3% (95% CI: 18.2 to 26.4) after adjustment for patient age, sex and the effect of clustering of patients within hospitals (Fig. 2). Hospitals were ordered in Figs 1 and 2 by ascending COPD readmission rate within 30 days.

### Sensitivity analysis

A sensitivity analysis was carried out to assess the potential impact of deaths in the community after discharge on risk of readmission for COPD. An adjusted COPD readmission rate within 90 days was calculated using an estimated 90-day mortality rate in the community of 25%. Twenty-five percent of 16,080 patients (those not readmitted within 90 days) represented 4020 additional patients who would have been at risk of readmission but who may not have been readmitted due to death unknown to the study. The current study’s COPD readmission rate within 90 days of 17.8% was applied to these 4020 additional patients, which would have provided an additional 716 COPD readmissions within 90 days. The adjusted total number of patients readmitted with COPD within 90 days in this sensitivity analysis was 716 + 3471 = 4187 patients. The adjusted COPD readmission rate within 90 days was 21.4%.

## Discussion

### Main findings

Of 20,932 patients in London (population 7.5 million) admitted to hospital with COPD 32% (6295) were readmitted within one year between 2006 and 2010. Ten percent were readmitted within 30 days and 18% within 90 days. Rates of COPD readmissions within 30 days and within 90 days did not vary among the majority of hospitals. Age and patient geographical deprivation score (IMD) were very weak predictors of readmission within 30 days or within 90 days. Adjustment for out-of-hospital mortality, or for day of the week of admission to or discharge from hospital did not influence readmission risk.

### Interpretation of findings in relation to previously published work

The low rates of COPD readmission within 30 days and within 90 days suggest that interventions designed to reduce the risk of COPD readmission have a small opportunity for improvement. There is no indication as to which patients might benefit from such an intervention. Among COPD patients receiving recommended pharmacological treatment there is limited evidence that additional interventions influence the risk of readmission.^34^ This is supported by the limited variation in the rate of COPD readmissions among the majority of hospitals in the current study. The variation in readmission rates between hospitals is in keeping with national emergency admissions data from 150 English NHS hospitals.^41^ These data showed little variation in admission rates between the majority (80%) of hospitals. Previous work, contemporary with the current study, has reported high levels of overtreatment of patients with COPD in London.^35^ It is likely that patients in the current study of COPD readmissions were receiving similar treatment to that reported in London at the same time.

The difference we observed in the risk of readmission within 30 days between patients whose LOS was 3–5 days compared to those whose LOS was ≤2 days was statistically significant but we doubt its clinical importance since the percentage difference between the groups was only 1%. We feel the same argument holds true regarding the difference observed in the risk of readmission within 90 days.

The COPD readmission rates reported in this study were in keeping with international findings.^30–33^ They were approximately half those reported by the RCP’s national audits of COPD care between 1997 and 2015.^29^ The accuracy and representativeness of the RCP audits’ reports of COPD readmissions have been questioned in this journal.^42^ In the RCP audits patients admitted to hospital with COPD were identified prospectively but had to have a known diagnosis of COPD at the point of admission.^29^ Patients in whom the diagnosis of COPD was not known at admission, even if that diagnosis was corrected to COPD subsequently during the admission were excluded. This method risked skewing enrolment towards those patients at the most severe end of the COPD spectrum. In addition, it is unlikely that those patients admitted for <1 day would have been included in the audits due to the practicalities of recruitment.

In contrast the study reported here provided a comprehensive picture of COPD admissions from a representative UK population. It included all admissions and readmissions from all COPD patients registered at London general practices, including readmissions to hospitals which were not the initial admitting hospital. As a result, the readmission rate in this study would have been predicted to be even higher than that of the RCP audits. This was not the case. Previous work which examined the LOS of COPD patients during their index admission to hospital, found that the mean interval between the index admission and first readmission was just under 400 days,^43^ a figure in keeping with the readmission rates reported here.

### Strengths and limitations of this study

The main strength of this study lies in the comprehensiveness of the data included. All NHS COPD admissions of patients registered at London general practices to all hospitals in England were included. This represents a population of at least 7.5 million people in which more than 97% were registered with general practices. The data relate to four consecutive years within a period in which we have previously demonstrated stable national rates of admission for COPD.^22^

Our data were complete for the population of London. They are unlikely to be representative of the whole of England. The recorded prevalence of COPD in London at the time of this study was 1.1% compared to 1.7% nationally so it may be that the risk of COPD is lower in London.^35^ Risk of admission for COPD in London is similar to the average risk nationally.^44^ Similarities between our data and those reported in New Zealand and the United States of America suggest our data reflect a risk of readmission for COPD in keeping with the disease in several different environments. Data were not available on a number of patient characteristics that may be related to risk of admission including COPD severity, patient performance status, socio-economic deprivation, treatment (pharmacological and non-pharmacological) received, and comorbidities.

### Implications for future research, policy and practice

This paper offers important implications for future policy, practice, and research in the management of COPD. The opportunity for intervening to prevent COPD readmissions is likely to be less than was previously thought because of the lower than expected rates of readmission of COPD we have found. We have shown that patients at risk of readmission cannot easily be identified using routinely available demographic data or differences in LOS or other medical care data. There is limited variation in the risk of COPD readmission between hospitals. This should discourage reliance on COPD readmission risk as a marker of quality of care. There is a need for better understanding of the role of disease severity in the risk of admission for COPD. Finally, a prospective study should be carried out to assess the impact of smoking cessation on preventing hospital admission and readmission in patients with established COPD who have moderate to very severe COPD.

## Conclusions

This study has provided new insights into the distribution of readmissions among patients admitted to NHS hospitals with COPD exacerbations. The finding of lower rates of COPD readmission than was previously estimated and the limited variation between hospitals calls into question the value of COPD readmission rate as a marker of quality of care. The potential savings resulting from improvements in COPD readmission rates, if they could be achieved, may not justify the cost of initiatives aimed at cutting those readmissions. Hospital level factors have not been demonstrated to be key determinants of COPD readmission. This suggests that patient level factors including disease severity may have a greater role in influencing readmissions for COPD. It emphasises the need to direct efforts towards smoking cessation in the primary and secondary prevention of COPD if we are to reduce the admissions and readmissions caused by it.

## Methods

### Study setting

London, the setting of this study, has an ethnically and socioeconomically diverse population of approximately 7.5 million people.^45^

### Study design and data source

This was a retrospective longitudinal observational study of all COPD patients registered at London general practices who had emergency NHS COPD hospital admissions between 1 April 2006 and 31 March 2010. The NHS Information Centre provided routinely collected data from the Hospital Episodes Statistics (HES) dataset, an inclusive record of NHS hospital activity within England.^46, 47^ HES track every hospital admission in the NHS providing publicly accessible data. Each HES record contained anonymised (but uniquely identified) information relating to an emergency hospital admission including patient demographics, Index of Multiple Deprivation (IMD) score for the patient’s address and registered general practitioner, diagnostic and procedural codes, admitting hospital, admission and discharge dates, discharge destination and LOS. IMD is a multi-dimensional score based on decennial national census data and annual local authority population data reflecting socio-economic deprivation within a geographical area based on the residential address of the patient.^48^ The diagnosis field was set to identify all patient admissions with a primary diagnosis of COPD (ICD-10 codes J40-J44) for the first episode within the hospital admission spell (the whole admission) between 2006 and 2010.

### Patient and hospital inclusion criteria

Analysis of COPD readmission rate was carried out for all patients who underwent an NHS COPD admission between 1 April 2006 and 31 March 2009 and who were aged ≥45 years at the time of that admission. Admission history prior to 1 April 2006 was not included in the analysis. The exclusion of younger patients was consistent with the criteria used in clinical trials of treatments for COPD.^49^ Younger patients may differ from older patients with COPD in their disease pathology and natural history.^50^ The majority of COPD patients are over 45 years. The cut off of 31 March 2009 for index cases was set so that any admissions in the subsequent year, up to and including 31 March 2010, of each patient so identified would be included in the data set.

NHS hospitals were identified by their provider codes and were included in the analysis if they had a record of ≥100 COPD patient admissions during the study period. This criterion was applied to allow a comparison of hospitals which had responsibility for emergency admissions whose medical care might have expected to include COPD. Hospitals specialising in operative procedures for COPD including bronchial stenting, bullectomy, lung volume reduction and transplantation were excluded. If two or more hospitals had merged during the study period, the admissions from each of the hospitals were combined. These mergers usually occurred between neighbouring hospitals. This meant that patients within the catchment areas of the two hospitals would have had a similar likelihood of being admitted to either hospital.

### Statistical analysis

#### Descriptive analysis

Descriptive analysis of the index COPD admissions was undertaken. This included analysis of the proportion of patients admitted and discharged on a weekday, the proportion of patients admitted for <1 day and a description of the LOS of the index COPD admissions. Weekday was defined as Monday to Friday, and weekend as Saturday or Sunday.

A comparison was made of the age, sex, and deprivation scores of patients readmitted with COPD within 1 year of the index COPD admission and those patients who were not readmitted within 1 year. The differences between the groups were analysed using the Chi-squared test and the independent samples *t*-test.

To examine the COPD readmission rate to hospital within 1 year of the index COPD admission, the proportion of patients readmitted on the same day, within 30 days, within 90 days, within 182 days and within 1 year among patients aged ≥45 years was calculated.

#### Sensitivity analysis of COPD readmission rate

The risk of death in the community among patients discharged after an admission for COPD was unknown. Those patients who died in the community would have been removed from the pool of patients at risk of readmission. A sensitivity analysis was performed to allow for the effect of unknown deaths among discharged patients after their index admission for COPD. An estimate of the proportion of deaths in the community was made using Office for National Statistics (ONS) rates of death in 2008, the midpoint of the study.^51^ The risk of readmission was recalculated on the assumption that these deaths had not occurred. The death rate within 90 days of admission for COPD has been recorded as between 13–17%.^52^ The 2008 ONS mortality figures listed annual death rates for patients aged 90 years and over as 241.3/1000 population (males) and 225.3/1000 population (females).^51^ For the current study an estimated 90-day mortality rate in the community of 25% was chosen. This was considered as a “worst-case scenario”. An adjusted COPD readmission rate within 90 days was calculated as an example.

#### Determinants of COPD readmission risk

To determine the influence that patient characteristics had on COPD readmission risk, two-tailed partial correlations were sought between mean patient age and mean patient IMD score per admission and the percentage of patients readmitted with COPD within 30 days and the percentage of patients readmitted with COPD within 90 days.

To determine the influence that LOS of index COPD admission had on the risk of readmission with COPD within 30 days and within 90 days, multiple logistic regressions were undertaken, controlling for patient sex, age, and deprivation score. LOS of each index COPD admission was grouped by quartile.

The influence on the risk of readmission with COPD within 30 days and the risk of readmission within 90 days of the day of the week on which the index COPD admission began and the day of the week on which it ended was determined. Chi-squared tests were performed to compare the risk of readmission if the index COPD admission had occurred on a weekday compared to at the weekend and to compare the risk of readmission if the discharge from the index COPD admission had occurred on a weekday compared to at the weekend.

To examine the influence that the hospital to which patients were admitted during their index COPD admission had on the risk of readmission with COPD to any hospital, the proportion of patients who had a COPD readmission within 30 days and within 90 days was compared between the 29 included hospitals, following adjustment for patient age, sex and the effect of clustering of patients within hospitals. A fixed effects model was used with each hospital entered as a separate term in the multiple logistic regression model as well as the above confounders. Hospitals were anonymised in the results.

The statistical packages SPSS version 20 (IBM Corporation, New York) and STATA version 13 were used for data analysis. Ethics approval was not required for this study which used routinely collected anonymised data to inform delivery of care.
